# UPANets: Learning from the Universal Pixel Attention Neworks

**DOI:** 10.3390/e24091243

**Published:** 2022-09-04

**Authors:** Ching-Hsun Tseng, Shin-Jye Lee, Jianan Feng, Shengzhong Mao, Yu-Ping Wu, Jia-Yu Shang, Xiao-Jun Zeng

**Affiliations:** 1 Department of Computer Science, The University of Manchester, Manchester M13 9PL, UK; 2 Institute of Management of Technology, National Chiao Tung University, Hsinchu 300, Taiwan; 3 School of Software, Yunnan University, Kunming 650504, China

**Keywords:** computer vision, image classification, CNN, attention

## Abstract

With the successful development in computer vision, building a deep convolutional neural network (CNNs) has been mainstream, considering the character of shared parameters in a convolutional layer. Stacking convolutional layers into a deep structure improves performance, but over-stacking also ramps up the needed resources for GPUs. Seeing another surge of Transformers in computer vision, the issue has aroused severely. A resource-hungry model is hardly implemented for limited hardware or single-customers-based GPU. Therefore, this work focuses on these concerns and proposes an efficient but robust backbone, which equips with channel and spatial direction attentions, so the attentions help to expand receptive fields in shallow convolutional layers and pass the information to every layer. An attention-boosted network based on already efficient CNNs, Universal Pixel Attention Networks (UPANets), is proposed. Through a series of experiments, UPANets fulfil the purposes of learning global information with less needed resources and outshine many existing SOTAs in CIFAR-{10, 100}.

## 1. Introduction

The development of computer vision has experienced a range of trends in this decade. Several introducing models [1,2,3,4,5,6] in open datasets competition significantly improved the accuracy of the image classification, which includes deep convolutional neural networks (CNNs) with residual calculation [7,8,9,10,11,12,13,14]. With the deep CNNs from stacking convolutional layers, models could capture local characteristics and global profiles with the increasing receptive fields [15]. However, this deep policy will raise the needed parameters and then makes one customer-based GPU unable to hold it. Besides CNNs, vision in Transformer (ViT) [16] has opened a path of applying the pure Multi-Head Attentions, which is from the natural language processing, to classify images by learning global information. While ViT arouses even influential works [17,18], we are facing a more severe issue of draining GPUs than deep CNNs because most Transformer-based networks require more powerful GPUs with large exclusive CUDA memory to calculate. Although sparse Attention in Informer [19] is trying to ameliorate the burden on GPUs, training Transformer-based models in a customer-based GPU to gain a decent performance remains impractical. It thus motivates this work to find a balance between computational costs and capturing image information globally.

**Figure 1:**
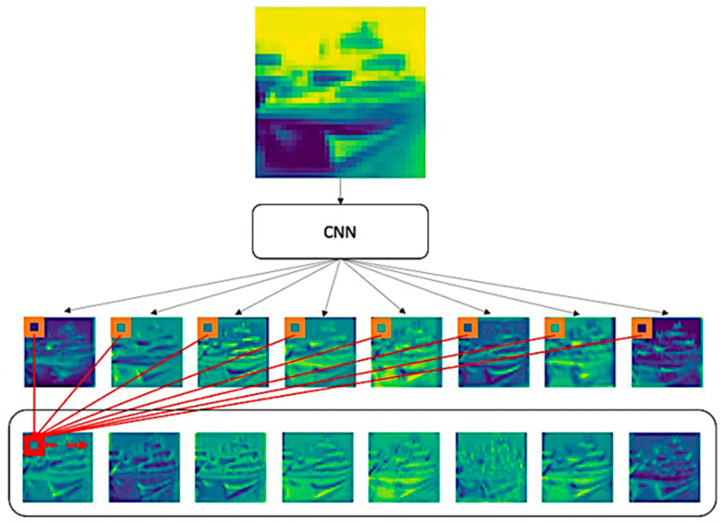
Channel Pixel Attention (CPA) Process and Samples. The image on the top is an originally sampled image from CIFAR-10. The feature maps in the middle line are the outputs generated from the CNNs before processing CPA. On the bottom line are the samples generated from CPA. The red square is the sum of weighted pixels from each orange square pixel in the same position.

To unleash the calculating pressure in a GPU and have a decent performance simultaneously, making a layer in shallow depth equipped with a broad receptive field is critical. Namely, if we can make layers mature quickly (in early depth), it is not necessary to have a deep structure to increase receptive fields. Instead of choosing already power-hungry Transformer-based structures, endowing learning global information ability to CNNs is rational because of the sharing filters mechanism in convolutional layers. Then, the issue that needs to be addressed is how to endow learning global information ability to CNNs rather than stacking convolutional layers to increase the receptive field. This work proposes Channel Pixel Attention (CPA) for helping convolutional layers to obtain global information directly, as shown in Figure 1. By CPA, models can combine information across the channel to generate more complex feature maps and further make shallow depth layers process similarly to deep depth layers.

In line with the same notion and the observations in [20], another direction to boost model learning and help information transport well among convolutional layers is building connections among a block, which is usually a unit with stacking of multiple convolutional layers and stacking as a layer module. To amplify the CPA effect, a hybrid connection with CPA is proposed. UPA blocks process lossless information from concatenating multiple UPA blocks in a stack and filter out vital information through residual connection. By this operation, the received information is not only from the last block but also the accumulating information until this block, so the CPA in each block can further absorb features from other blocks to amplify the receptive fields.

To transmit lossless learned information and feedback among layers, connecting each layer module is common in many object-detection tasks (e.g., saliency, semantic, and instance objects detection) within an auto-encoder-based structure in which the input and output share the exact image size with a bottleneck in a network. Nevertheless, the same picture is not seen in image classification. In contrast, the effect of merely applying residual and concatenating connections to prevent information loss will eventually saturate [21]. For this reason, the Extreme Connection (ExC) is proposed to connect each UPA layer module with learnable Spatial Pixel Attention (SPA) along with the existing Global Average Pooling in UPANets. As a result, instead of simply extending the connection to classification, a learnable global pooling in spatial direction, SPA, involves making sure that essential pixels occupy a significant portion in the sending of information to the output layer. A smooth updating landscape is expected to be generated to ensure robustness.

Thus, by forming the proposed components into balanced but powerful networks, we furtherly propose Universal Pixel Attention Networks (UPANets). Through the evaluations of this work, the contributions can be summarized as follows:

- Propose UPANets with UPA Block equipped with Channel Pixel Attention (CPA) and a combination of Spatial Pixel Attention (SPA) and Extreme Connection (ExC) connecting each UPA Layer Module to find the balance between performance and cost.
- CPA considers pixel information across channels and helps CNNs form complex features even in shallow depth with fewer parameters. In addition, by applying concatenating feature maps in a network, the capturing ability of CPA can be amplified to cross blocks detection.
- SPA and ExC help to generate a smooth learning landscape and contribute to learning spatial information to pass important pixel information, respectively.
- A competitive image classification model surpasses well-known SOTAs in CIFAR-{10, 100} and Tiny ImageNet.

This paper firstly discusses the essential background and motivations in Section 1, and then the well-known observations and relevant works toward image classification are introduced in Section 2. Next, the core of this work, the proposed method UPANets and its structures are introduced in Section 3. Then, to examine the proposed method, the comparisons between the proposed methods, UPANets, and other novel methods in well-known datasets can be observed in Section 4. Finally, the conclusion is in Section 5, and the extra findings and simulations of UPANets are summarized in the Appendix A, Appendix B, Appendix C and Appendix D. We share our implemented code at the link: https://github.com/hanktseng131415go/UPANets (accessed on 17 July 2022).

## 2. Related Works

### 2.1. Attentions

Attention exists in many forms. Motivated by Transformer [22], Multi-Head Attention served the purpose of considering global information, so the model becomes robust and powerful, but the draining computational resource breaks the balance between performance and used parameters. Although ViT [16] used the image patches to reduce the needed parameters, the problem remains. Later, the variants [17,18] with multiple Transformer-based units in a network make the draining problem severer. Apart from learning a mega dataset to make Transformer’s Attentions useful, DeiT-B [23] used the proposed attention to transfer the pre-trained parameters into a Transformer on image classification. However, it only relocates the draining issue from end-to-end training to knowledge distillation. Despite this dilemma, big companies, such as Google, Microsoft, and Facebook, do not stop exploring more because of the path of considering global information in Transformers. Nonetheless, having a stack of GPUs is not common for most users, which drives the need to learn global information with fewer resources.

On the ground of applying Attentions in CNNs, the draining issue caused by Attentions is minor, but the existing Attentions mainly focus on local information or are limited toward paying attention in the current block. One of the most well-known Attention in CNNs is Convolutional Block Attention Module (CBAM) [24], which arbitrarily applies max and average pooling to care pixels. Although the Attentions in CBAM are parameter-less, the potential of losing information by max and average pooling remains. Similarly, SENets [25] uses global average pooling to squeeze the spatial information into one representative value. Then, it uses multi-layers perceptron (MLP) with a ReLU and Softmax to make channel attention. By embedding SE-Block after each block afterword, it showed improvements in VGGs [2], InceptionNets [3,4], and ResNeXts [26], but the same issue of losing information from arbitrarily average pooling is hovering. In object detection, operation toward a convolutional output to serve the purpose of pixel attention is also a trend. For example, DANet [27] is embedded behind a backbone network as a feature extractor (e.g., ResNets [5]) and applies two dot-products on the outputted feature maps with Softmax among the two Attentions in the Channel Attention Module, and the same operation on the width and height of feature maps in the Position Attention Module. However, these modules only care about the feature maps from the final layer module. Additionally, GCNet [28] uses a similar module as DANet’s in each block and replaces the dot-product operations with a 1×1 convolutional kernel, namely paying attention to the current block. The applying operation in GCNet with a 1×1 convolutional kernel did not outshine using a one-layer perceptron as a cross-channel attentional operation in our comparison in Appendix B. Another attention to learning cross channels in the current block is the shuffle operation from ShuffleNets v1 [29] and v2 [30]. Shuffling the order of CNNs kernel weight in groups breaches the independent learning process when detecting images in group CNNs, so the next layer of group CNNs can detect the other group CNNs feature maps. Nonetheless, the shuffle operation is taken back, and thus performance is limited; and please see Section 4.2.2. Unit Subtraction Convolution (USC) in RK-Net [31], similar to the parameter-free operation in ShuffleNets, replaces conventional dot-product with subtraction to extract the key points from the feature map. Another replacing traditional convolutional operation work is Local Pattern Network (LPN) [32], which proposes a feature partition strategy to take advantage of contextual features with the parameter-free operation. By viewing ShuffleNets, USC, and LPN, colouring CNNs operation with different mechanisms help the network to consider more information to perform better. In sum, inheriting a similar notion, our CPA brings a new direction to pay attention to across the channel. The mature feature maps are furtherly proved with better performance compared with the 1×1 convolutional layer, shuffle operation, and SENet in Section 4.

### 2.2. Structure Design

A good structure design could affect the performance and the parameters convert ratio because of being able to help information to transmit to different layers properly. ResNets have introduced residual connection that offers a great path to let deep learning fulfil the true meaning of deep, namely letting original information pass to deep layers intact. Additionally, the residual connection prevents the potential of facing overfitting. To explain the underlying reasons, the visualization of the loss landscape [20] has proven that. Another underlying reason in [20] is the dense connection in DenseNets [33]. Densely connection connects original and outputting information by reusing feature maps in deep layers. By observing landscapes from DenseNets in [20], the loss landscapes are smoother than ResNets. However, according to the statement from EfficientNet [21], the effect will be saturation despite using skip-connection or densely-connection. Most importantly, in our simulation of Section 4.3, the efficiency between parameters and accuracy degrades severely when layers in both ResNets and DenseNets grow. In our comparison in Section 4.3, UPANets has a better conversion ratio than the formers.

## 3. UPANets

This section details the proposed method, UPANets, and the relevant components. Firstly, the Attention approach designed channel-wise is revealed. Consequently, a hybrid block with residual and concatenation learning in UPA Block shows how they work together in UPANets. After the proposed SPA in ExC, the structure of UPANets is shown.

### 3.1. Channel Pixel Attention

A convolutional kernel is good at capturing local information, but each kernel can only detect a specific pattern, which limits the ability. Conventionally, stacking convolutional layers to expand the pattern library is intuitive but deadly, as discussed in Section 1. To expand the library without overstacking layers, having the ability to learn global information is vital. Therefore, the Channel Pixel Attention (CPA) is proposed. CPA applies a one-layer perceptron to pay attention to the pixel in the same position across channels. By fusing patterns across channels, the library can be expanded, and the detected patterns can also be more complex; please see Figure 1. The operation can be represented as (1):

(1) $$X=\overset{n}{\underset{c=1}{\sum }}x_{c}^{R}W_{c}^{T}+b,$$

where c indicates the *c*th channel, $X\in {\mathbb{R}}^{N\times C\times W\times H}$, $x_{c}^{R}\in {\mathbb{R}}^{N\times W\times H\times C}$, which is reshaped to perform a dot product with $W_{c}^{T},$ $W_{c}^{T}\in {\mathbb{R}}^{N\times C\times C}.$

**Figure 2:**
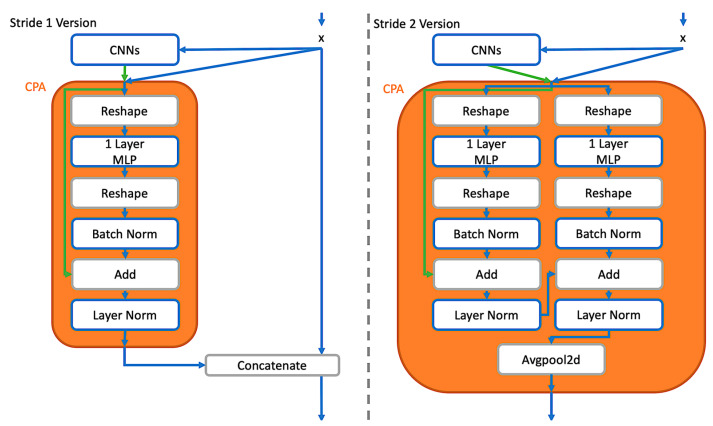
Channel Pixel Attention Structure. The green and blue lines represent the original and processed information, respectively. In the stage of stride one, one CPA is involved with a concatenation. In the stage of stride two, a parallel CPA are processed with residual learning to decide essential to pass, and then a down-sampling is applied by avgpool2d.

After the pixel attention is processed by a one-layer perceptron, Batch Normalization and Layer Normalization with residual connection are applied afterwards. The workflow of CPA can be demonstrated in Figure 2, and the sample feature maps toward the inputs in an actual image with demonstration are shown in Figure 1, in which the outputted feature maps from the CPA process combine their original features and supportive information from others. These combined features show that CPA can promote feature maps to fuse more complex ones without losing original features. Compared with the deep structure, CPA can help a shallow network form more complex patterns, expanding receptive fields.

### 3.2. UPA Blocks

**Figure 3:**
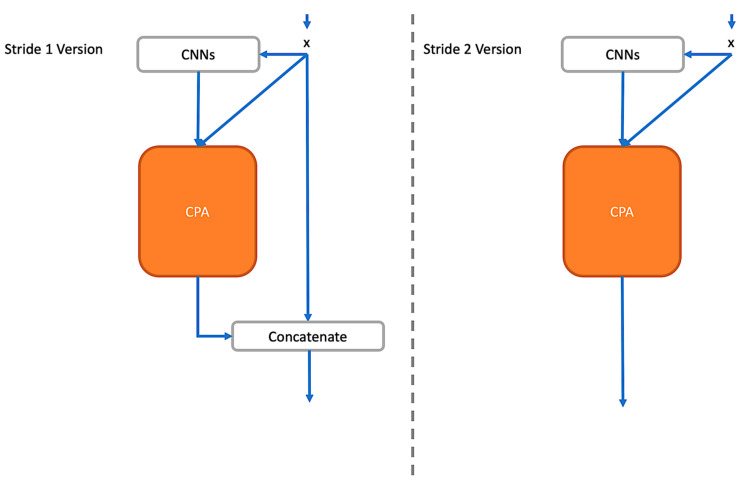
UPA Blocks Structure in the Stride One and Stride Two Sets.

As the discussion in Section 2 toward ResNets and DenseNets, it is crucial that a block not only processes image information well but can also pass lossless features not to waste the processed information from previous blocks. To achieve that, a hybrid combination is proposed for collecting the feature maps from the previous blocks by concatenating and filtering out essential feature maps to the next layer by residual learning. By concatenating, it can preserve original information and further help to amplify the CPA effect by learning not only the cross channels information in the current block among UPA layer modules, see Section 3.3. Furtherly, please, see the UPA Blocks structure in Figure 3.

Observing Figure 2 and Figure 3, the difference between stride one and stride two is whether to use the concatenate operation or not. On the other hand, the residual connection is applied in CPA, which determines whether it should output the current learned information or the ones from the last block.

### 3.3. UPA Blocks

**Figure 4:**
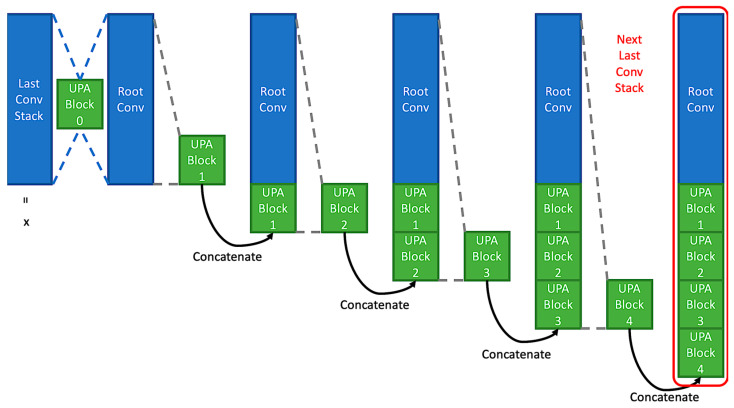
UPA Layer Module. In the UPA block 0, a stride two UPA block using the residual connection with 2×2 kernel average pooling is applied.

Continuing the discussion in Section 3.2, a model can preserve processed information from previous blocks by applying concatenation in UPA Block. After that, combining multiple UPA Blocks to form a layer module makes CPA pay attention to the vital pixel across channels from multiple blocks. Namely, through CPA, CNNs can access every processed feature in the layer module. If it comes to down-sampling, a parallel residual CPA involves deciding the important pixel from the accumulating feature maps to pass. See Figure 4; UPA Layer Module helps CPA pay attention across channels throughout accumulating blocks.

In Figure 4, except for the stride two version operation in block 0, each block follows the stride one version operation. Further, the width of every stride one version block is smaller than its input shape, and that can be referred to in the following Equation:

(2) $$w_{b}=W_{l}/b,$$

where b=1⋯n, $W_{l}$ indicates the summation of adding width of this layer, the width is the filter number or channel number, $w_{b}$ means the outputted width of this block, and $w_{0}$ equals to double width of the last layer because the original input remains, and the processed information is appended after that. For example, if the width of layer module 1 is set to 16, the outputted width of layer module 1 will be 32 because of concatenation. Therefore, the width of block 0 in layer module 2 is 32, $w_{0}=32$. Then, as the number of blocks in layer 2 is 4, b=4, the width of each block is 8, $w_{b}=8$ because $W_{0}=32$ and $\frac{32}{4}=8$. In this case, the outputted width from this UPA Block of the current UPA Layer Module will be 32+8=40.

### 3.4. Spatial Pixel Attention

Although Global Average Pooling (GAP) does not require extra computational cost, it is suffering the potential of losing information because of arbitrarily averaging out overall spatial information. To ameliorate this concern, this work proposes Spatial Pixel Attention (SPA) with learnable parameters by applying a one-layer perceptron to learn essential pixels in the same spatial direction. With the involved learnable process, SPA helps to determine which pixel to be amplified or ignore. SPA mechanism can be defined as the following formula:

(3) $$X=\overset{n}{\underset{c=1}{\sum }}x_{c}^{R}W_{c}^{T}+b,$$

where c indicates the *c*th channel, $X\in {\mathbb{R}}^{N\times C\times 1}$, $x_{c}^{R}\in {\mathbb{R}}^{N\times C\times L}$, L=W×H, and $W_{c}^{T}\in {\mathbb{R}}^{N\times L\times 1}$.

**Figure 5:**
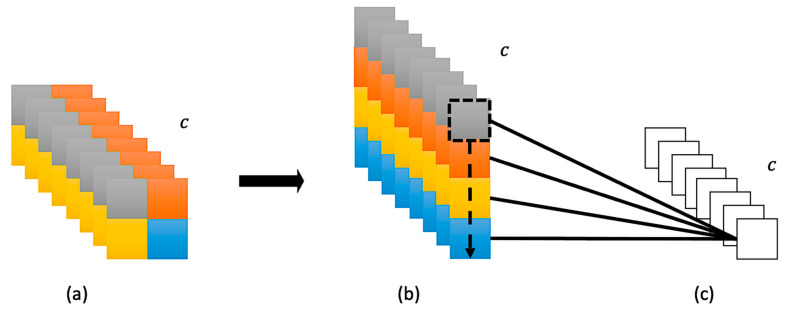
Spatial Pixel Attention. The demonstration takes a 2×2 feature map, as shown in (**a**), with c=8 as an example. Then, the process from (**a**) to (**b**) reshapes the convolutional image. From (**b**) to (**c**) is the SPA process, and its function is similar to the global average pooling.

In Figure 5, the process from (b) to (c) is implemented by a one-layer perceptron. Through the layer, SPA can determine to pay the appropriate attention to the essential pixels and then squeeze the entire pixels into one-pixel information by a dot-product instead of arbitrary pooling with average.

### 3.5. Extreme Connection

**Figure 6:**
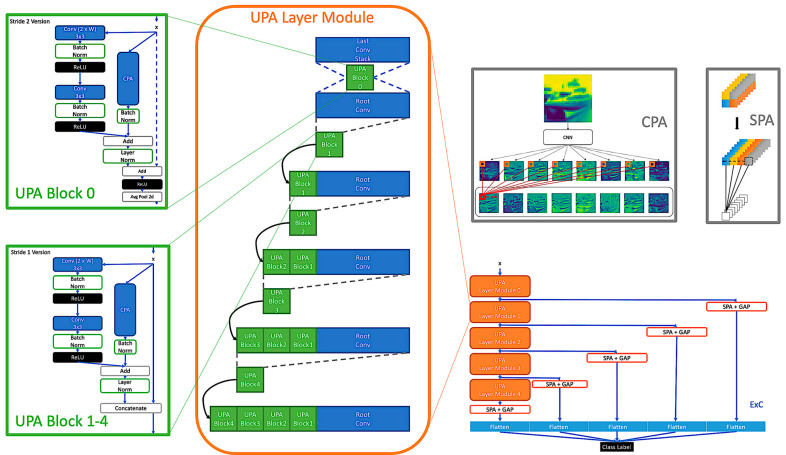
Structure of UPANets. The illustration of the proposed modules is assembled by showing the ExC among UPANets in the bottom right corner.

Connecting the output layer with each inner layer in a network often generates a smooth landscape [20]. With a smooth landscape, the probability of having a robust result with many merits, such as quickly converging, arises. To do that, building such a connection would help. Here, an Extreme Connection (ExC) is proposed, which considers both the information from SPA and GAP. Figure 6 reveals the applied extreme connection, and this operation can be represented as the following:

(4) $$X=F[SPA_{1}\left(x_{1}^{R}\right)+GAP_{1}\left(x_{1}^{R}\right),\;\cdots ,\;SPA_{b}\left(x_{b}^{R}\right)+GAP_{b}\left(x_{b}^{R}\right)],$$

where $X\;\in \;{\mathbb{R}}^{N\times C}$, which is the output from the flatten-concatenate F. N is the data number, and C represents the number of channels. Additionally, b means the block^th^ in a network.

As shown in Figure 6, ExC builds the relationship from the final hidden layer to the output of each block. In addition, SPA evaluates which pixel should be paid more attention toward the class to support GAP. Integrating both operations with layer normalization allows both sides’ information to be scaled to the same level to learn.

### 3.6. UPANets Structure

In Figure 6, the cooperation between each proposed module is illustrated. The proposed CPA is applied among each UPA Block. Additionally, ExC is applied to connect every UPA Layer Module with the proposed SPA to cooperate with GAP. The detail transferring of size, width, and the proposing Attention in UPANets toward CIFAR-10 is presented in Table A1.

## 4. Experiment

### 4.1. Experiment Environment Settings

This simulation implemented UPANets and is compared with CNNs-based SOTA models. The experimental environment comprises a customer-based GPU (RTX Titan with 24 GB) and an eight-core CPU (intel i9-9900KF) with 32 GB RAM. Despite the limitation of available hardware, although we cannot implement ImageNet to evaluate, this simulation experiment compared UPANets and others in CIFAR-{10, 100} and tiny ImageNet datasets. Every training process was implemented in a cosine annealing learning schedule with a half cycle. Additionally, the training optimizer was stochastic gradient descent with an initial learning rate = 0.1, momentum = 0.9, and weight decay = 0.0005. A simple combination of data argumentation was applied with random crop in padding = 4, random horizontal flip, normalization in CIFARs and tiny ImageNet. As this simulation conducted a series of experiments with different epochs, the specific number of used epochs is revealed before each sub-section experiment description.

On the other hand, apart from mainly recording performance in accuracy (Top—1 Error), because we argue that finding a balance between performance and used resources is essential, efficiency is applied to examine the turnover rate throughout the experiments. This consideration shows that blindly chasing higher performance by adding parameters is irrational. The efficiency can be represented as the following Equation:

(5) E=Acc/P,

where E represents the efficiency, P means the size of used parameters, and Acc is the abbreviation of the accuracy. Through Equation (5) above, it can learn whether this structure or setting can convert the parameters into performance efficiently, and it can also be recognized as the ratio of accuracy and parameters. For example, if two parameters contribute a 100% accuracy, the efficiency could be presented as E = 0.5. Additionally, if four parameters contribute another 100% accuracy, the efficiency could be presented as E = 0.25. Following the above examples, E = 0.5 is greater than E = 0.25, meaning higher efficiency.

### 4.2. Ablation Study

In this sub-section, we implemented a series of ablation comparisons toward different components among UPANets. The performance of UPANets with F = 16 in CIFAR-{10, 100} are revealed in the following comparisons, as “F” shows in Table A1, and each performance was recorded in the testing stage with the highest accuracy. The total number of epochs in this sub-section was set to 100, and the experiment setting followed the previous description in Section 4.1.

#### 4.2.1. Global Fusion from Channel Pixel Attention

By Section 3.1, it is expected that CPA can promote CNNs to consider the global information of images as ViT [22], but CPA achieve that by only conducting a one-layer perceptron. By this one-layer perceptron, CPA only requires one-third of parameters compared with the Attentions in ViT with processing a Query, Key, and Value from three one-layer perceptrons every time.

**Figure 7:**
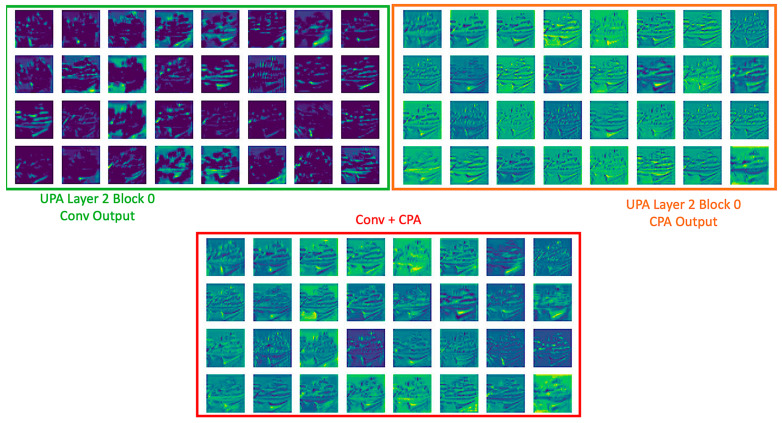
Samples of Fusion Feature Maps in UPANets.

In order to illustrate learned global information from CPA, Figure 7 is sampled from the first 32 feature maps from the CNNs in UPA Block 0 before the CPA in UPA Layer 2. Figure 7 contains three rectangles in green, orange, and red. It is evident that the green region from CNNs only detected a specific pattern of the kernel, and some kernels only detected background information. However, a feature map remains dim if the kernel cannot detect a feature. Most importantly, based on the concatenation in UPA Block and operation in the UPA layer module, although residual and concatenation are involved, CNNs still only detected specific patterns. A typical way to prevent dull outputs is adding more width to increase the pattern variety in CNNs, but it is a curse to ramp up more parameters. The above discussion explains the saturation of ResNets and DenseNets, despite residual and concatenation learning.

Conversely, with the help of CPA, the orange area is immune to the issue in CNNs. Additionally, with UPA Blocks and UPA Layer Modules, the green region of the first 16th feature maps is from the root CNNs (CNNs in UPA Layer Module 0), 17th to 20th feature maps are from the root and UPA Block 0, etc. By CPA seeing feature maps from root CNNs to UPA Block 4, the outputs from CPA are gradually complex. That shows the capability of learning cross channels global information block to block and helps to expand the receptive field directly. Therefore, every feature map from CPA covers the learned information from itself to the others, so each pixel considers pixels located at the same position as others by learnable weights. Namely, the CPA can determine which pixel is helpful for consideration. Lastly, the samples of Conv + CPA possess the detected local patterns from the CNNs and conclude the global features from others. A sample of learned patterns in CNN and CPA by inputting noise can be seen in Appendix C.

#### 4.2.2. Comparing with ShuffleNet

When we look at learning global information, it can be understood as sharing learned information with others. Under this notion, as the discussion toward ShuffleNets in 0, the shuffle operation is close to this idea. By shuffling the order of independently learned feature maps, the afterwards grouped CNNs have the chance to map to the pattern from the different groups. The groups in CNNs are dividing the channels (filters) into several independent groups to detect (e.g., channels = 16, groups = 2, which means they will be separated into two groups with 8 channels where each group will not share the learned parameters). Therefore, in Table 1, a comparison between CPA and the shuffle operation in ShuffleNets is evaluated under CNNs groups in two and four, respectively. The bold font indicate the best performance in the indicator.

#### 4.2.3. Building Connection with Learnable Pooling

In Section 3.5 toward ExC, one of the reasons for introducing the connection is creating a smooth loss landscape to raise the potential for having a robust result. To verify this idea, the best approach is plotting the landscapes from loss and Top–1 Error (accuracy). Therefore, a series of landscape visualizations toward models with and without ExC in CIFAR-10 is conducted followingly. Additionally, we argued that arbitrarily GAP spatial information would suffer with the potential of losing important information. As a result, along with the visualizations, the proposed SPA participated in this simulation with performance evaluations in CIFAR-{10, 100} afterwards.

**Figure 8:**
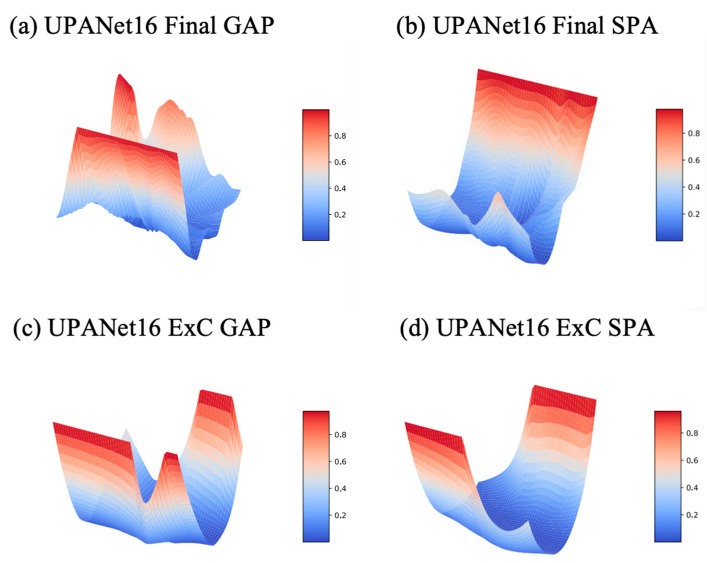
Scaled Loss Landscapes of UPANets16 Variants.

**Figure 9:**
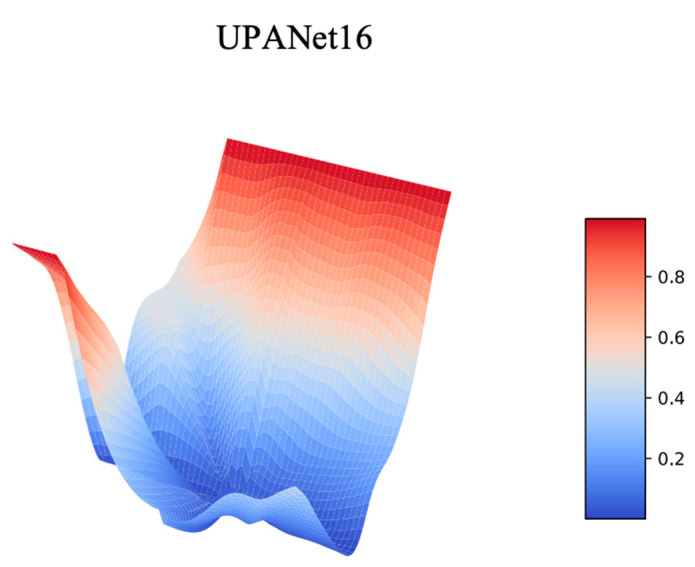
Scaled Loss Landscapes of UPANet16.

**Figure 10:**
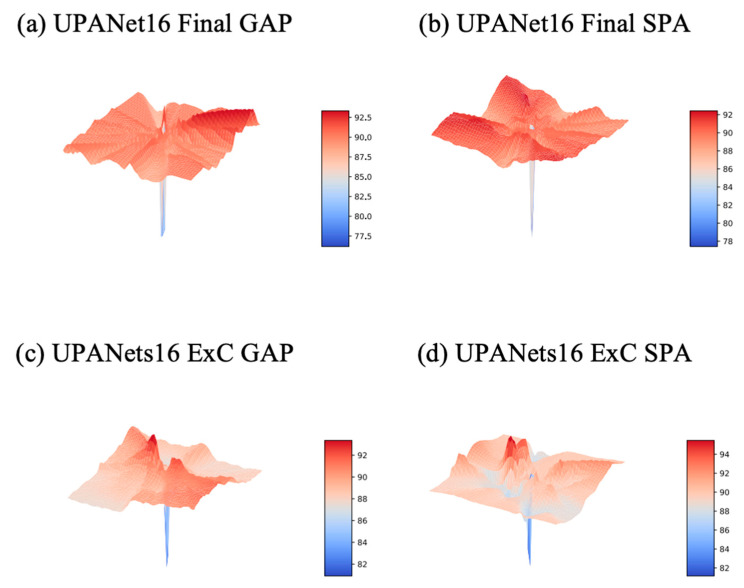
Top—1 Error Landscapes of UPANets16 Variants.

**Figure 11:**
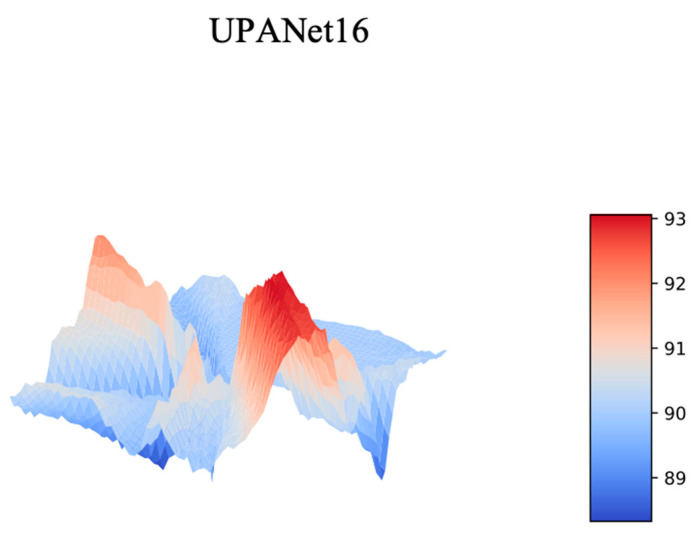
Top—1 Error Landscapes of UPANets16.

On the ground of visualizing landscapes, to make the loss of each competitor the same, we applied a min-max scaler to scale each loss into [0:1], and then we could compare the landform under the same standpoint. For Top—1 Error, as the scale is already in [0:1] in percentage, the scaling is skipped toward accuracy. Please see the landscapes toward scaled loss and Top—1 Error from Figure 8 to Figure 9 and Figure 10 to Figure 11, separately.

Regarding the benefits of SPA, we have seen that it helps smooth the landscape. Another observable benefit, in Table 2, is performance boosting. Although, compared Final SPA with Final GAP, the performance increased in both CIFAR-{10, 100}, the winnings are reversed when cooperating with ExC. Whereas the non-absolute improvement of working with ExC, the improvement happened while ExC and GAP worked together. A more significant improvement is also seen in having ExC, SPA, and GAP together, in the bold fonts. Given the most significant improvements in performance and landscapes, we opt for ExC + SPA + GAP with proposed methods to form UPANets.

### 4.3. Comparison with SOTAs

After evaluating a range of proposed components, these vital parts form UPANets, and it is vital to compare them with existing SOTAs. In UPANets, setting F = 16, 32, and 64 as the channel number base represents different widths of UPANets. Using these variant width UPANets with existing CNNs-based models in CIFAR-{10, 100} as former simulations, we can see a much clearer place among SOTAs. Additionally, because of the hardship of being unable to evaluate on ImageNet, a Tiny ImageNet is chosen as an alternative. In the following comparison, the models are reimplemented based on the work in the link (https://github.com/kuangliu/pytorch-cifar accessed on 23 October 2020) following the experiment setting in Section 4.1, except for setting epochs in 200.

#### 4.3.1. Comparison in CIFARs

**Figure 12:**
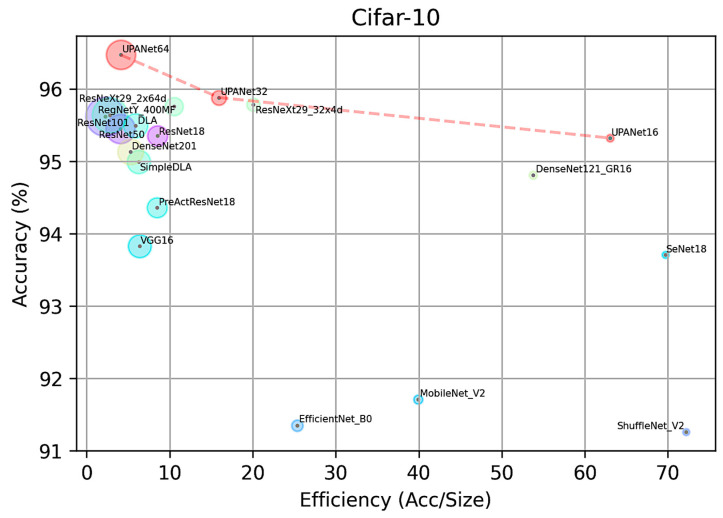
UPANets Performance Position with SOTAs in CIFAR-10.

**Figure 13:**
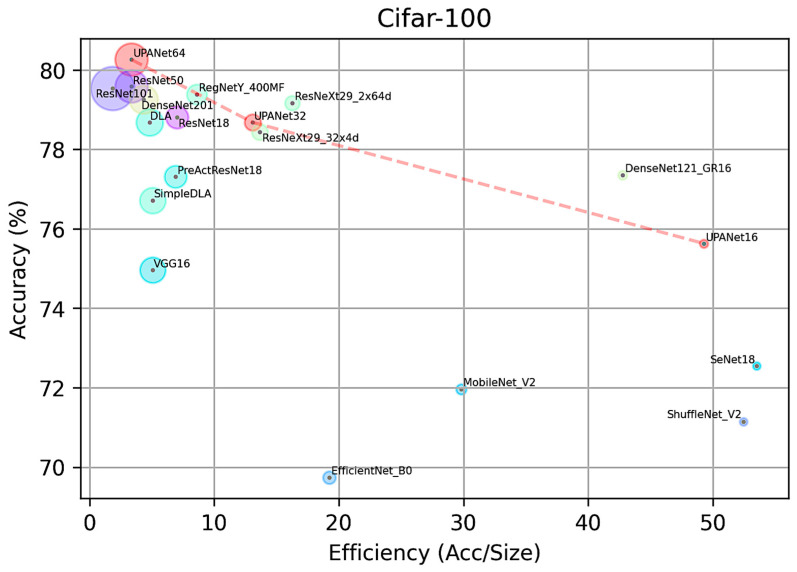
UPANets Performance Position with SOTAs in CIFAR-100.

In this comparison, the performance of each model was recorded in accuracy toward testing data, parameters size in million, and efficiency in Equation (5) with the best performance in the bold fonts in tables. As there are three performance indexes in Table 3, it presents the information in a scatter plot as Figure 12, which contains accuracy on the *y*-axis and efficiency on the *x*-axis. The size of the circle toward each model represents the parameter size in a million. The same policies apply to Table 4 and Figure 13.

In this implemented CIFAR-10 comparison, UPANet64 has the best accuracy. By plotting each model in Figure 12, UPANets have outstanding performance-balancing efficiency and accuracy in the scatter plot. In addition, the models claimed in the lite structure are located in the bottom right area, but they lost certain accuracy. On the other side, UPANet16 and DenseNet are located in the upper right corner, indicating that the proposed model and DenseNets have high efficiency. As for the accuracy in Table 3, UPANet64 is the only model reaching over 96% accuracy without many parameters, especially compared with ResNet101 and DenseNet201. A similar overall distribution toward the three indexes is witnessed in implemented CIFAR-100 comparison. Although UPANet16 and UPANet32 are falling behind in terms of efficiency, UPANet64 is the one which passes the 80% accuracy in CIFAR-100. As a result, UPANets performed well in both open datasets from the evaluated points.

#### 4.3.2. Comparison in Tiny ImageNet

Although we compared a series of SOTAs with UPANets in CIFAR-{10, 100}, the difficulty of datasets is smaller than Tiny ImageNet, as it needs to classify more labels, which is about double that of CIFAR-100. Moreover, the image size is two times larger than CIFARs, so we only examined UPANets64 in 100 epochs with the same experimental setting as the above comparisons. Further, some SOTAs, which were also examined on Tiny ImageNet, are shown together in Table 5.

As a whole, UPANets has not only performed excellently in widely-used datasets but also in a complex dataset, in this Tiny ImageNet. Moreover, based on classification performance, the proposed UPANets can be one of the state-of-the-art models in the Tiny ImageNet benchmark (Checked on April 2021).

## 5. Conclusions

This work proposed a novel backbone, UPANets, for image classification. Each proposed component in the framework fulfils specific objectives and helps the model outshine existing SOTAs in terms of performance and efficiency. The positive findings and potential contributions can be concluded as follows.

### 5.1. CPA in Processing Global Information with Benefits

First, CPA captures global information across channels to form more complex feature maps, expanding the receptive fields of shallow layers. That is, the shallow layers will quickly mature to boost performance. On the other hand, the more mature layers indicate fewer needs for stacking deep. With further application of concatenation in UPA blocks with accumulating UPA layer modules, the effect is amplified more to ramp up the advantages.

### 5.2. SPA with ExC Brings Better Environments for Learning

Connecting each layer, transporting essential spatial information by learnable attention brings smoother landscapes. As the concern of losing information by arbitrarily averaging out spatial pixels, SPA ameliorates it with performance improvements. Moreover, ExC learned that passing feedback from SPA to each layer forms a smooth landform.

### 5.3. SPA with ExC Brings Better Environments for Learning

Finally, comparing with a series of SOTAs in CIFAR-{10, 100} and Tiny ImageNet, the results of UPANets are better than most existing SOTAs. As a result, it is convinced that UPANets can perform competitively in image classification. Further, this practical evidence shows that learning universal pixels channel-wise and spatial-wise with the proposed modules can effectively utilize parameters.

In sum, these attempts create a way to develop an efficient backbone for effectively processing universal information with decent performance.

## Figures and Tables

**Table 1:** Comparison of Cross Channels Learning in UPANet16.

UPANet16	CIFAR-10 Acc % (Top 1 Error)	CIFAR-100 Acc % (Top 1 Error)	Size (Million) (CIFAR-10, CIFAR-100)	Efficiency (Acc % / Million) (CIFAR-10, CIFAR-100)
w/o CPA w/o Shuffle CNNs in groups = 1	93.54 (0.0646)	72.98 (0.2702)	(1.43, 1.48)	(65.32, 49.42)
w/o CPA w/o Shuffle CNNs in groups = 2	92.64 (0.0736)	71.0 (0.29)	(0.72,0.77)	(129.67, 92.21)
CPA CNNs in groups = 2	94.2 (0.058)	74.52 (0.2548)	(1.02, 1.06)	(92.53, 70.12)
Shuffle CNNs in groups = 2	93.33 (0.0667)	71.98 (0.2802)	(0.96, 1.01)	(96.75, 71.31)
w/o CPA w/o Shuffle CNNs in groups = 4	90.69 (0.0931)	68.75 (0.3125)	(0.37, 0.41)	**(245.11, 167.68)**
CPA CNNs in groups = 4	93.79 (0.0621)	73.55 (0.2645)	(0.78, 0.83)	(119.58, 88.73)
Shuffle CNNs in groups = 4	92.93 (0.0707)	71.33 (0.2837)	(0.73, 0.78)	(127.14, 91.97)
CPA CNNs in groups = 1 (Ours UPANet16)	**94.90 (0.0510)**	**75.15 (0.2485)**	(1.51, 1.56)	(62.85, 48.17)

**Table 2:** Comparison of UPANets16 Variants in CIFAR-{10, 100}.

UPANet16	CIFAR-10 Acc % (Top 1 Error)	CIFAR-100 Acc % (Top 1 Error)	Size (Million) (CIFAR-10, CIFAR-100)	Efficiency (Acc %/Million) (CIFAR-10, CIFAR-100)
Final GAP	94.66 (0.0534)	74.63 (0.2537)	(1.507162, 1.530292)	(63.11, 48.78)
Final SPA	94.70 (0.0530)	74.72 (0.2491)	(1.506427, 1.530306)	**(63.13, 48.84)**
ExC + GAP	94.75 (0.0525)	74.60 (0.2540)	(1.510042, 1.554772)	(62.75, 48.13)
ExC + SPA	94.60 (0.0542)	75.09 (0.2491)	(1.512431, 1.557161)	(62.64, 48.45)
ExC + SPA + GAP	**94.90 (0.051)**	**75.15 (0.2485)**	(1.512431, 1.557161)	(62.85, 48.71)

**Table 3:** UPANets Performance with SOTAs in CIFAR-10.

Model	Test Avg Accuracy	Size (M)	Efficiency
ShuffleNet_V2	91.26	1.26	**72.21**
EfficientNet_B0	91.35	3.60	25.38
MobileNet_V2	91.71	2.30	39.93
SeNet18	93.71	1.34	69.69
VGG16	93.83	14.73	6.37
PreActResNet18	94.36	11.17	8.45
DenseNets121_16GR	94.81	1.76	53.78
SimpleDLA	94.99	15.14	6.27
DenseNet201	95.13	18.10	5.25
UPANet16 (Ours)	95.32	1.51	63.13
ResNet18	95.35	11.17	8.53
ResNet50	95.45	23.52	4.06
RegNetY_400MF	95.46	5.71	16.71
DLA	95.49	16.29	5.86
ResNet101	95.62	42.51	2.25
UPANet32 (Ours)	95.88	5.93	15.93
ResNeXt29_2x64d	95.76	9.13	10.49
ResNeXt29_32x4d	95.78	4.77	20.06
UPANet64 (Ours)	**96.47**	23.60	4.09

**Table 4:** UPANets performance with SOTAs in CIFAR-100.

Model	Test Avg Accuracy	Size (M)	Efficiency
EfficientNet_B0	69.74	3.63	19.22
ShuffleNet_V2	71.15	1.36	52.47
MobileNet_V2	71.96	2.41	29.83
SeNet18	72.55	1.36	**53.49**
VGG16	74.96	14.77	5.07
SimpleDLA	76.72	15.19	5.05
UPANet16 (Ours)	76.73	1.56	49.05
preactresnet18	77.31	11.22	6.89
DenseNets121_16GR	77.35	1.81	42.76
RegNetY_400MF	78.44	5.75	13.64
DLA	78.68	16.34	4.82
UPANet32 (Ours)	78.78	6.02	12.90
ResNet18	78.81	11.22	7.02
ResNeXt29_32x4d	79.16	4.87	16.27
DenseNet201	79.25	18.23	4.35
ResNeXt29_2x64d	79.38	9.22	8.61
ResNet101	79.54	42.70	1.86
ResNet50	79.59	23.71	3.36
UPANet64 (Ours)	**80.29**	23.84	3.37

**Table 5:** UPANets Performance with SOTAs in Tiny ImageNet.

Model	Test Avg Accuracy	Size (M)	Efficiency
DenseNets + Residual Networks [34]	60.00	N/A	N/A
PreActResNets18 [35]	63.48	N/A	N/A
UPANets64 (Ours)	**67.67**	24.40	2.77

**Figure A1:**
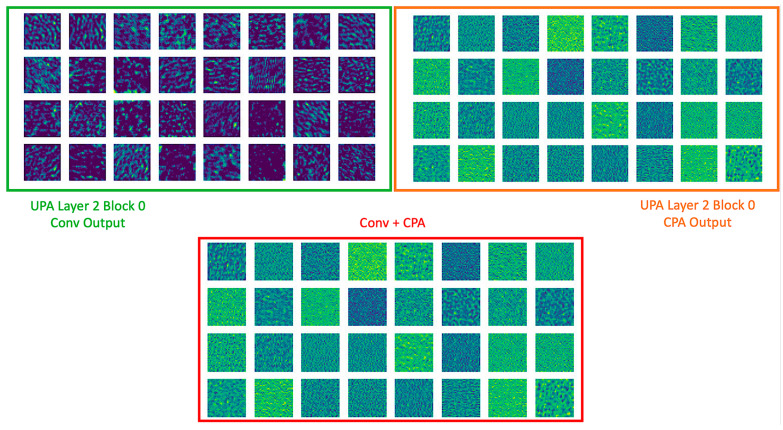
Samples of Fusion Feature Maps in UPANets with Noise.

**Figure A2:**
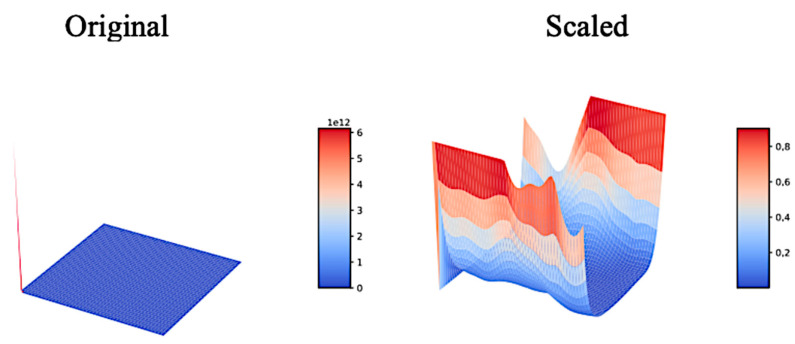
The Pre and Post Scaled Loss Landscape of DenseNets.

**Figure A3:**
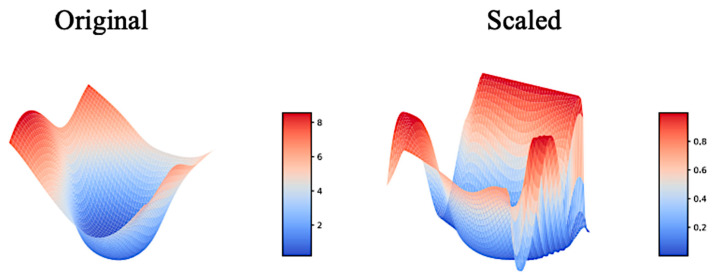
The Pre- and Post-Scaled Loss Landscape of UPANets16.

**Figure A4:**
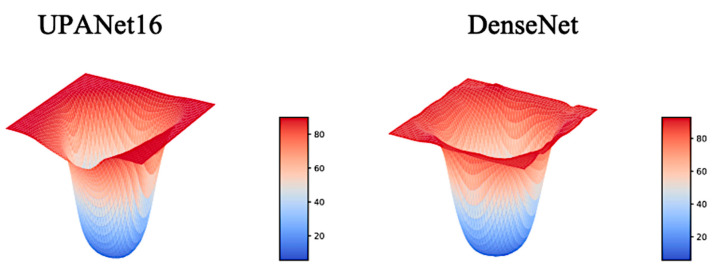
The Top—1 Error Landscapes of UPANets16 and DenseNet.

**Figure A5:**
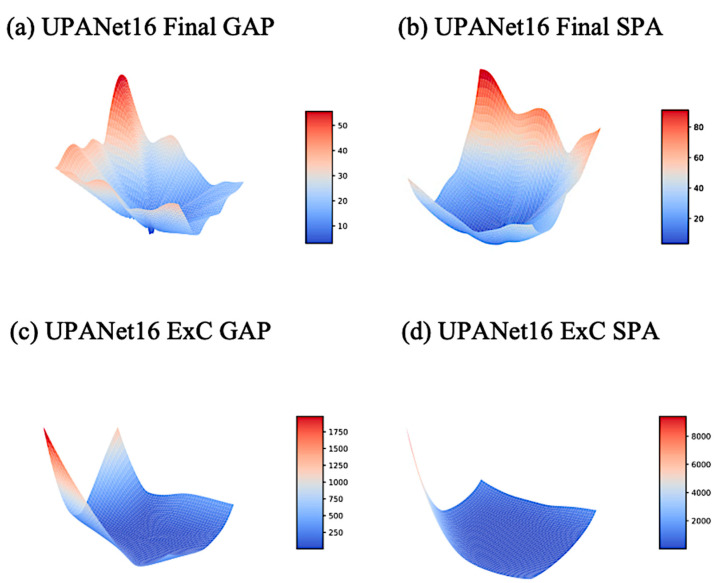
Original Loss Landscapes of UPANets16 Variants.

**Figure A6:**
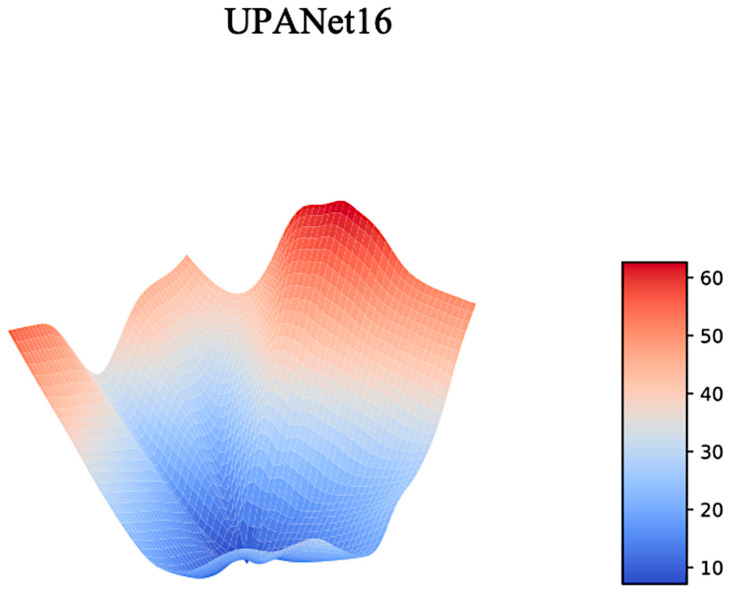
Original Landscapes of UPANets16.

## Data Availability

This research is analyzed based on CIFAR-{10, 100} datasets (from https://www.cs.toronto.edu/~kriz/cifar.html accessed on 23 October 2020) and Tiny ImageNet (from https://www.kaggle.com/c/tiny-imagenet accessed on 23 October 2020).

## Appendix A. Dimension Illustration in UPANets Structure

In the Table A1 for UPANets structure in CIFAR-10, N represents the data number, F indicates the filters number, $B_{i}$ are blocks, d means the depth multiplier, b is the number of the block, and w is the convolutional width. UPA Block 0 and the other blocks follow the stride 2 version and stride 1 version UPA block, respectively.

**Table A1:** The UPANets Structure for CIFAR-10.

Layers	Blocks	Input size	Output size	Structure
UPA Layer Module 0	UPA Block 0	N×32×32×3	N×32×32×F	$\left\{\left[\begin{array}{c} 3\times 3\;conv,\;2w\\ 3\times 3\;conv,\;1w \end{array}\right]+CPA\right\}$
UPA Layer Module 1 $B_{1}=4d$	UPA Block 0	N×32×32×F	$N\times 32\times 32\times {\left(\left(\frac{F}{B_{1}}\right)+F\right)}_{F_{0}}$	$\left\{\left[\begin{array}{c} 3\times 3\;conv,\;2w\\ 3\times 3\;conv,\;1w \end{array}\right]+CPA\right\}$
	UPA Block 1~4d	$N\times 32\times 32\times F_{b-1}$	$N\times 32\times 32\times {\left(\left(\frac{F}{B_{1}}\right)+F_{b-1}\right)}_{F_{b}}$	$\left\{\left[\begin{array}{c} 3\times 3\;conv,\;2w\\ 3\times 3\;conv,1w \end{array}\right]+CPA\right\}\times 4d$
UPA Layer Module 2 $B_{2}=4d$	UPA Block 0	N×32×32×2F	$N\times 16\times 16\times {\left(\left(\frac{2F}{B_{2}}\right)+2F\right)}_{F_{0}}$	$\left\{\begin{array}{c} \left[\begin{array}{c} 3\times 3\;conv,2w\\ 3\times 3\;conv,1w \end{array}\right]+CPA,\\ Avgpool2d\left(stride\;2\right) \end{array}\right\}$
	UPA Block 1~4d	$N\times 16\times 16\times F_{b-1}$	$N\times 16\times 16\times {\left(\left(\frac{2F}{B_{2}}\right)+F_{b-1}\right)}_{F_{b}}$	$\left\{\left[\begin{array}{c} 3\times 3\;conv,2w\\ 3\times 3\;conv,1w \end{array}\right]+CPA\right\}\times 4d$
UPA Layer Module 3 $B_{3}=4d$	UPA Block 0	N×16×16×4F	$N\times 8\times 8\times {\left(\left(\frac{4F}{B_{3}}\right)+4F\right)}_{F_{0}}$	$\left\{\begin{array}{c} \left[\begin{array}{c} 3\times 3\;conv,2w\\ 3\times 3\;conv,1w \end{array}\right]+CPA,\\ Avgpool2d\left(stride\;2\right) \end{array}\right\}$
	UPA Block 1~8d	$N\times 8\times 8\times F_{b-1}$	$N\times 8\times 8\times {\left(\left(\frac{4F}{B_{3}}\right)+F_{b-1}\right)}_{F_{b}}$	$\left\{\left[\begin{array}{c} 3\times 3\;conv,2w\\ 3\times 3\;conv,1w \end{array}\right]+CPA\right\}\times 4d$
Ex-Connected Layer		$\left[\begin{array}{c} \begin{array}{c} N\times 32\times 32\times F\left(\mathrm{Layer}\;\mathrm{Module}\;0\;output\right),\\ N\times 32\times 32\times 2F\left(\mathrm{Layer}\;\mathrm{Module}\;1\;output\right),\\ N\times 16\times 16\times 4F\left(\mathrm{Layer}\;\mathrm{Module}\;2\;output\right),\\ N\times 8\times 8\times 8F\left(\mathrm{Layer}\;\mathrm{Module}\;3\;output\right), \end{array}\\ N\times 4\times 4\times 16F\left(\mathrm{Layer}\;\mathrm{Module}\;4\;output\right), \end{array}\right]$	N×1×1×(F+F+4F+8F+16F)	SPA+ GAP
Output Layer		N×1×31F	N×10	Linear

## Appendix B. Comparison of Perceptron and CNNs in Attention

In Section 3.1, we bring a cross Channels Pixel Attention (CPA) mechanism. A one-layer perceptron is applied to offer the service in CPA. Additionally, 1×1 CNNs is a standard option to map the information across channels. However, as the simulation in Table A2 shows, the performances of CNNs have fallen behind using one-layer perceptron, one-layer perceptron in bold fonts. The underlying reason could be that although CNNs can share patterns, the single parameter in each sharing pattern is limited to carrying on vital information. Additionally, as the one-layer perceptron is operating in dot-product, the information is shared and combined with each one, indicating our point of CPA detecting cross channels in the same pixel position.

**Table A2:** The Comparison of Perceptron and CNNs as an Attention.

UPANet16	CIFAR-10 Acc % (Top 1 Error)	CIFAR-100 Acc % (Top 1 Error)	Size (M) (10, 100)	Efficiency (Acc %/M) (10, 100)
CNNs	94.76 (0.0442)	74.85 (0.2515)	(1.51, 1.56)	(62.75, 47.98)
FC	**94.90 (0.051)**	**75.15 (0.2485)**	(1.51, 1.56)	**(62.85, 48.17)**

## Appendix C. Sample Pattern of the CNN and CPA in UPA Block

CPA paying attention under the operation of UPA blocks among UPA layer modules, CPA can learn cross-channels-blocks pixel to form universal attention. That is contributed by concatenation in UPA blocks and accumulating of UPA layer module. To observe the learned patterns from the global range, inputting a random noise to extract pattern profiles was conducted in Figure A1, with the same extracting policy as Figure 7.

## Appendix D. Landscape toward UPANets and Others

The introduction of the visualizing loss landscape method in [20] helps researchers understand the possible training landscape among the parameters of a model. By the description of the actual implementing source code (https://github.com/tomgoldstein/loss-landscape, https://github.com/JoelNiklaus/loss_landscape accessed on 20 April 2021), the primary usage is setting a random sampling range in [−1:1] with a specific sampling number (default: 50). However, as this sampling method is similar to the sensitivity analysis in determining feature importance, only a good sampling range can produce a calculatable loss. This dilemma impeded us when we were trying to visualize a sensitive model, such as DenseNets, because a little adding noise might cause the loss to Nan. Therefore, how to define a good sampling range is a challenge. On the other hand, although filter normalization has been introduced [20] to compare loss landscapes from different models, we found that different loss ranges still make comparing hard. An enormous total loss range will make most landscapes smother because an outlier will break the harmony of the loss map.

Toward the dilemmas, we ushered an automatic search and min-max scaled into our visualization. First, a doable visualization range with binary search is applied in advance based on the original method from [−1:1]. Later, we used min-max scaling for every loss landscape to make the two landscapes comparable. Finally, for demonstrating, we end-to-end trained DenseNets and our models in CIFAR-10 based on the code in this project (https://github.com/kuangliu/pytorch-cifar accessed on 23 October 2020) and applied the ushered methods in the following pre- and post-scaled landscapes.

### Appendix D.1. Comparison with DenseNet

The visualizable sampling range was [−0.0375:0.0375] with 50 samples. In Figure A2, the largest loss broke the harmony of the original loss landscape. The normal loss owns the majority number, but it is hard to see the fluctuation of the landscape from the relative more minor loss. As a result, a flattened space created an illusion. Min-max scaled loss landscape shows a much different view. Although the centre of the map is still flat, the surrounding loss stands erect on edge. Not only can the scaled landscape reveal a much more reasonable profile, but scaling can also make different landscapes comparable. Therefore, the exact search and scaled policies were applied to UPANet16 in [−0.0375:0.0375] to compare with DenseNet in Figure A3 and the Top—1 Error ones in Figure A4.

Comparing Figure A2 to Figure A4 in the range [−0.0375:0.0375], UPANet can distribute a similar view as DenseNet, but there are fewer enormous losses at the edge of the landscape. Especially, it seems models can quickly reach a minimum in UPANet16 Top—1 Error map with a lower gap in the margin.

### Appendix D.2. UPANet16 Variants Original Landscapes

In our visualizations in UPANet16 and its variants, using the default range of [−1:1] can already offer the visualization, which indicates UPANets are not as sensitive as DenseNets. This also implies the robustness of UPNets toward the noise, as the method of [20] is sampling parameters from a different angle, like adding noise to see the loss changing. So, the sensitive changes formed the landforms we obtained. The scaled ones have been shown in Section 4.2.3. Figures from Figure A5 to Figure A6 show the pre-scaled landscapes for each variant of UPANet16.
